# Assessing the Origin of and Potential for International Spread of Chikungunya Virus from the Caribbean

**DOI:** 10.1371/currents.outbreaks.2134a0a7bf37fd8d388181539fea2da5

**Published:** 2014-06-06

**Authors:** Kamran Khan, Isaac Bogoch, John S. Brownstein, Jennifer Miniota, Adrian Nicolucci, Wei Hu, Elaine O. Nsoesie, Martin Cetron, Maria Isabella Creatore, Matthew German, Annelies Wilder-Smith

**Affiliations:** Department of Medicine, Division of Infectious Diseases, University of Toronto, Toronto, Canada; Department of Medicine, Division of Infectious Diseases, University of Toronto, Toronto, Canada; University Health Network, Divisions of Internal Medicine and Infectious Diseases, Toronto, Canada; Boston Children’s Hospital, Harvard Medical School, Boston, Massachusetts, USA; Li Ka Shing Knowledge Institute, St. Michael's Hospital, Toronto, Canada; Li Ka Shing Knowledge Institute, St. Michael's Hospital, Toronto, Canada; Li Ka Shing Knowledge Institute, St. Michael's Hospital, Toronto, Canada; Children’s Hospital Informatics Program, Boston Children’s Hospital, Boston, Massachusetts, USA ; Department of Pediatrics, Harvard Medical School, Boston, Massachusetts, USA ; Network Dynamics and Simulation Science Laboratory, Virginia Bioinformatics Institute, Virginia Tech, Blacksburg, Virginia, USA; Division of Global Migration and Quarantine, Centers for Disease Control and Prevention, Atlanta, USA; Departments of Medicine and Epidemiology, Emory University School of Medicine and Rollins School of Public Health, Atlanta, USA; Li Ka Shing Knowledge Institute, St. Michael's Hospital, Toronto, Canada; Li Ka Shing Knowledge Institute, St. Michael's Hospital, Toronto, Canada; Lee Kong Chian School of Medicine, Nanyang Technological University, Singapore; Institute of Public Health, University of Heidelberg, Germany

## Abstract

Background: For the first time, an outbreak of chikungunya has been reported in the Americas. Locally acquired infections have been confirmed in fourteen Caribbean countries and dependent territories, Guyana and French Guiana, in which a large number of North American travelers vacation. Should some travelers become infected with chikungunya virus, they could potentially introduce it into the United States, where there are competent Aedes mosquito vectors, with the possibility of local transmission. Methods: We analyzed historical data on airline travelers departing areas of the Caribbean and South America, where locally acquired cases of chikungunya have been confirmed as of May 12th, 2014. The final destinations of travelers departing these areas between May and July 2012 were determined and overlaid on maps of the reported distribution of Aedes aeygpti and albopictus mosquitoes in the United States, to identify potential areas at risk of autochthonous transmission. Results: The United States alone accounted for 52.1% of the final destinations of all international travelers departing chikungunya indigenous areas of the Caribbean between May and July 2012. Cities in the United States with the highest volume of air travelers were New York City, Miami and San Juan (Puerto Rico). Miami and San Juan were high travel-volume cities where Aedes aeygpti or albopictus are reported and where climatic conditions could be suitable for autochthonous transmission. Conclusion: The rapidly evolving outbreak of chikungunya in the Caribbean poses a growing risk to countries and areas linked by air travel, including the United States where competent Aedes mosquitoes exist. The risk of chikungunya importation into the United States may be elevated following key travel periods in the spring, when large numbers of North American travelers typically vacation in the Caribbean.

## INTRODUCTION

Chikungunya virus is a mosquito-transmitted alphavirus endemic to sub-Saharan Africa and South and East Asia. In recent years, chikungunya has been appearing outside of its endemic zone as a result of increasing international travel.^1^^,^ 2 Concurrently, the geographic ranges of *Aedes aeygpti* and *albopictus* - the primary vectors for chikungunya virus – have been expanding, a phenomenon thought to be a consequence of climate change and globalization.^3^ The combination of international travel by potentially infected persons and the increasing geographic availability of competent vectors has set the stage for the introduction and spread of Chikungunya to previously unaffected areas. In recent years, autochthonous transmission of chikungunya has occurred in non-endemic areas such as the 2007 outbreak in Italy and 2010 outbreak in France, and most recently, in multiple Caribbean Islands where competent *Aedes* mosquitoes exist.^1^

The geographic dispersion of chikungunya virus may occur in instances where susceptible travelers in endemic areas are bitten by infected female *Aedes *mosquitoes.^4^ After the typical incubation period of 3-7 days (range 2-12 days), infected individuals become viremic.^5^^,^ 6 Among those who develop illness, common symptoms include fever, headache, rash, and severe symmetrical polyarthralgia,. The potential for an infected individual to then transmit chikungunya virus to a susceptible *Aedes *mosquito is greatest during the first 2-6 days of illness, during the viremic phase.^7^

For the first time in the Americas, chikungunya was reported among non-travelers on the Caribbean island of St. Martin in December 2013.^8^ Since then, locally acquired cases have been reported in multiple countries and territories in the region for a total count of over 4,000 probable or confirmed cases, raising concerns that this virus could spread into and within neighboring areas, including parts of the United States.^9^^,^ 10

Every year, large numbers of North American tourists vacation in the Caribbean during spring and summer months. After returning home, these individuals could potentially introduce chikungunya virus into areas where the conditions necessary for autochthonous transmission exist. We used a novel approach combining a number of datasets related to travel routes, volumes of travelers, historic temperature data and zoonotic distribution of *Aedes* mosquitoes in order to model the recent outbreak in the Caribbean and the risk of spread to other countries via international travel. Due to the large travel volume between the Caribbean and the U.S. we conducted an analysis to determine the vulnerability of U.S. cities and states to the importation of chikungunya virus and subsequent local transmission due to favorable environmental conditions.

## METHODS

We accessed anonymized, worldwide, passenger-level flight itinerary data for 2012 from the International Air Transport Association (IATA). The IATA dataset represents an estimated 93% of the world’s commercial air traffic at the passenger level. Flight itinerary data includes information on the airport where the traveler initiated their trip, and where relevant, connecting flights leading up to their final destination.

Using this dataset, we first analyzed the origins of all air travelers departing chikungunya endemic areas of the world (as defined by the U.S. Centers for Disease Control and Prevention)^11^ that had final destinations in the Caribbean region (as defined by the United Nations)^12^ during the period from October to December 2012 (to assess potential origins of chikungunya virus introduction into the Caribbean in December 2013).

Next, we analyzed the final international destinations of all travelers (between May and July 2012) departing areas of the Caribbean where locally acquired cases of chikungunya have been confirmed as of May 12th, 2014 (i.e. Aruba, Anguilla, Antigua, British Virgin Islands, Dominica, Dominican Republic, French Guiana, Guadeloupe, Haiti, Martinique, St. Barthelemy, St. Kitts and Nevis, and St. Martin, Sint Maarten, St. Vincent and the Grenadines).

We then calculated the volume of travelers departing these indigenous areas of the Caribbean between May and July 2012 with and their countries of final destination. We also calculated city-level volumes of travelers with final destinations in North America. These monthly city-level travel data were mapped and overlaid with the geographic extents of *Aedes aeygpti *and *Aedes albopictus *mosquitoes across the United States.^13^ We then determined the average monthly temperature of each state between May and July using 60 years of historical data.^14^ While there are many unknowns regarding the climatic conditions necessary for *Aedes aeygpti *and* albopictus *mosquitoes to transmit chikungunya virus,^15^ an average temperature of 20° Celsius was identified as an important threshold in the 2007 chikungunya outbreak in Italy.^16^^-^ 18

## RESULTS

While the specific origin of the Caribbean chikungunya epidemic is not precisely known, we found that five countries were the source of 84.4% of all international air travelers departing chikungunya endemic areas of the world with final destinations in the Caribbean region between the months of October and December 2012. These countries included South Africa (4,348 travelers; 23.4% of all travelers from chikungunya endemic areas of the world), India (4,012 travelers; 21.6%), China (2,561 travelers; 13.8%), Philippines (2,555 travelers; 13.7%) and the French territory of Réunion (2,218 travelers; 11.9%).

With respect to the possibility of receiving an imported case via international air travel, the final destinations of travelers departing areas of the Caribbean where locally acquired cases of chikungunya have been confirmed (as of May 12th, 2014), over the three-month period from May to July 2012 are shown in Table 1. Three countries represented the final destinations of 70.0% of all travelers worldwide. The United States, including Puerto Rico, had the strongest links through international air travel (1,071,658 travelers; 52.1% of the global total), followed by France (298,921 travelers; 14.5%), and the Netherlands Antilles, not including Sint Maarten (68,604 travelers; 3.3%). By comparison, ten cities represented the final destinations of 49.0% of all travelers. These included New York (283,224 travelers; 13.8% of the global total), Paris (240,204 travelers; 11.7%), Miami (161,430 travelers; 7.8%), San Juan, Puerto Rico (80,571 travelers; 3.9%), Curacao (48,594 travelers; 2.4%), Fort Lauderdale (45,076 travelers; 2.2%), Madrid (41,286 travelers; 2.0%), Boston (40,829 travelers; 1.9%), Toronto (36,162 travelers; 1.7%), and Caracas (29,973 travelers; 1.4%).

**Table 1: Leading Destination Countries for Travelers Departing Chikungunya Indigenous Areas of the Caribbean:** * Between May and July 2012 † Includes Puerto Rico

**Country**	**Traveler** Volume*	**Global Total (%)**	**Cumulative Total (%)**
United States†	1,071,658	52.2	52.2
France	298,921	14.5	66.7
Netherland Antilles	68,604	3.3	70.0
Canada	64,736	3.2	73.2
Spain	55,329	2.7	75.9
Venezuela	42,774	2.1	78.0
Germany	36,984	1.8	79.8
United Kingdom	28,480	1.4	81.1
Italy	27,159	1.3	82.4
St. Lucia	24,102	1.2	83.6
Panama	23,576	1.2	84.8

For the United States, the final city-level destinations of travelers for May, June and July are shown in Figures 1-3. The volume of travel to cities in the United States was generally greatest in July. Cities with the overall highest travel volumes included New York City, Miami and San Juan (Puerto Rico). Among the subset of cities where *Aedes aeygpti* or *albopictus *are present and where average temperatures are expected to approach or exceed 20° Celsius between May and July, the highest travel volumes were to San Juan, Miami, and Charlotte.

**Volume of Travelers from Chikungunya Indigenous Areas of the Caribbean* to the United States and Canada in May†:**
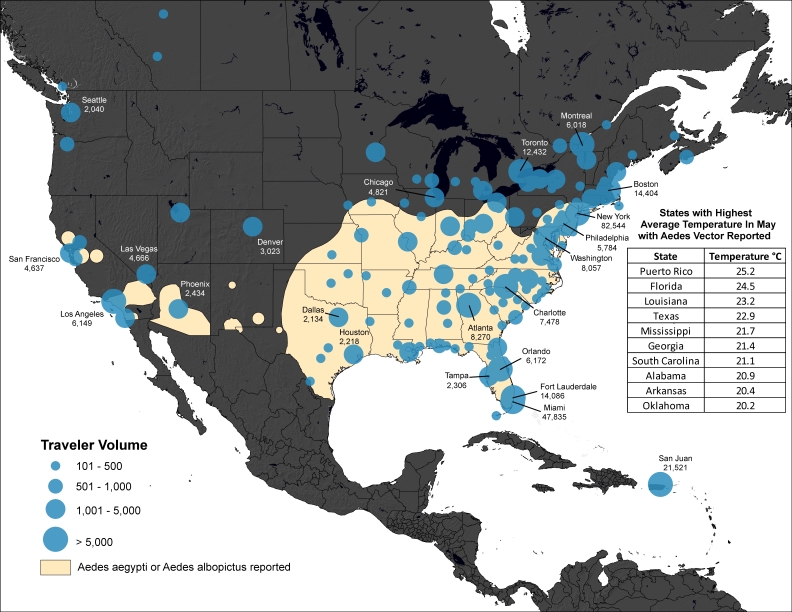
* As of May 12th 2014 † Using historic air travel data from May 2012

**Volume of Travelers from Chikungunya Indigenous Areas of the Caribbean* to the United States and Canada in June†:**
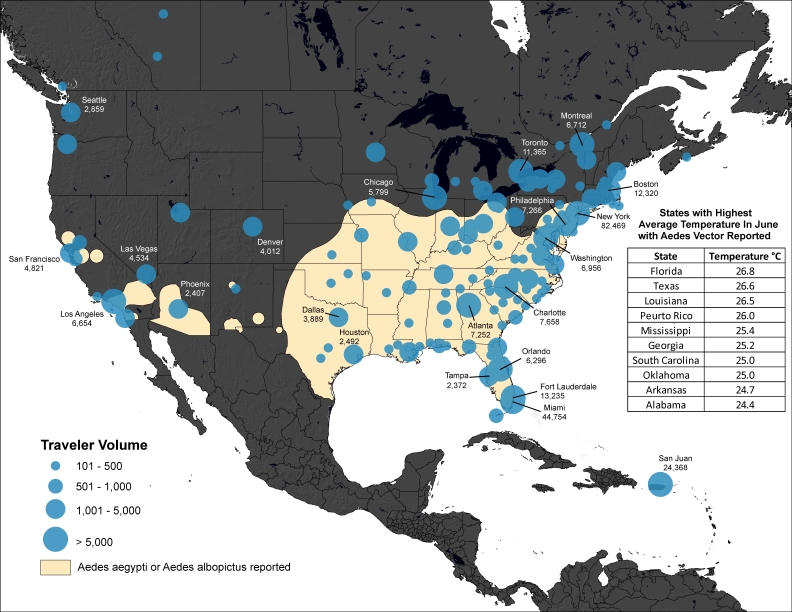
* As of May 12th 2014 † Using historic air travel data from June 2012

**Volume of Travelers from Chikungunya Indigenous Areas of the Caribbean* to the United States and Canada in July†:**
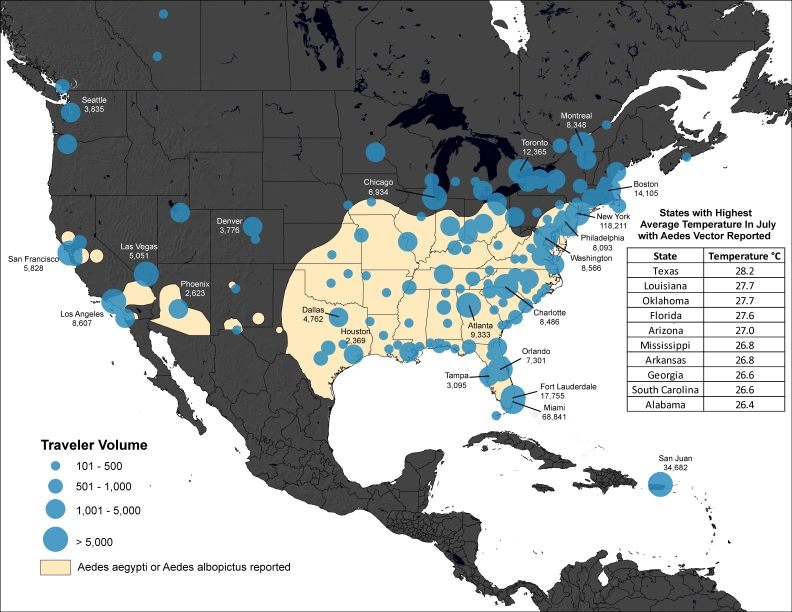
* As of May 12th 2014 † Using historic air travel data from July 2012

## DISCUSSION

Global forces from climate change to surging worldwide air travel are contributing to the globalization of vector-borne diseases such as West Nile virus, dengue and chikungunya.^18^^-^ 21 In December 2013, chikungunya virus was identified for the first time in the Americas, where it has since caused over four thousand locally acquired cases across numerous Caribbean islands in addition to the South American nations of Guiana and French Guiana. While the origins of chikungunya introduction in the Caribbean are not precisely known, molecular diagnostics have determined that the strain currently circulating in the region belongs to the subtype CHIKV-JC2012 and closely resembles a strain found in China, the Philippines and Micronesia.^22^ Our analysis suggests that five chikungunya endemic countries account for the vast majority of international air travel into the Caribbean region in the months leading up to the first reported cases, with China and the Philippines accounting for 27.5% of all such travelers. However, the probability of importation into the Caribbean is a function not only of travel volumes but also of chikungunya incidence in the origin countries.

Our analyses indicate that the United States is the final destination of over half of all travelers departing chikungunya indigenous areas of the Caribbean, followed by France, which accounts for almost 15% of all travelers. The United States has never reported local transmission of chikungunya virus, despite the presence of *Aedes aeygpti* and *albopictus* mosquitoes across the southeastern region of the country, while autochthonous transmission of chikungunya has previously been documented in southeastern France in 2010, where *Aedes albopictus* is known to exist.^23^ Furthermore, many North American travelers vacationing in the Caribbean will return to areas of the United States where the climate may be suitable for autochthonous transmission.

We found that New York City, Miami and San Juan are the leading U.S. destination cities of travelers from chikungunya indigenous areas of the Caribbean between May and July. Healthcare providers in these locations should familiarize themselves with the clinical presentation of chikungunya, which overlaps significantly with dengue fever. The early detection of chikungunya is particularly important in areas such as San Juan, Miami, and Charlotte where competent mosquito vectors could become infected through bites of viremic travelers.^9^ Symptomatic individuals with suspected or confirmed chikungunya infection should take special measures to avoid mosquito bites in the week following the onset of their illness (when viremia is greatest) to decrease the potential for autochthonous spread.

Although there are many unknowns about the biology of *Aedes* mosquitoes and the specific climatic conditions that would support autochthonous transmission, warmer weather is thought to shorten the interval between the time when an *Aedes* mosquito is infected by a viremic patient and when that mosquito can transmit the virus to another susceptible human host (i.e. the *extrinsic incubation period*).^24^ Since the 2013-2014 winter season has been unseasonably cool across many parts of the United States, this could favor longer extrinsic periods, and consequently a lower probability of viral transmission from vector to human host. Although belonging to a different strain from the one currently circulating in the Caribbean, of potential concern is the chikungunya E1-A226V mutation identified during the 2005-2006 Réunion epidemic, which facilitated more efficient transmission specifically in *Aedes albopictus* mosquitoes.^25^^,^ 26 This mutation was subsequently imported to Italy, and has since appeared in China and Papua New Guinea.^25^^,^ 27 ^-^ 29 However, this mutation does not appear to dominate in the major chikungunya outbreaks that occurred in India 2006-2010.^30^

Our analysis has several important limitations. First, we are relying on accurate identification of indigenous chikungunya cases in the Caribbean region to conduct our analyses of population movements through air travel. Some countries in the Caribbean may have limited infectious disease surveillance capacity, particularly for a newly emerging pathogen such as chikungunya. Our transportation analysis was also limited to commercial air travel despite the fact that many individuals vacationing in the Caribbean may travel on cruise ships or other means of transport. This limitation would presumably lead to an underestimate of travelers arriving in U.S. port cities that face the Caribbean islands, though the length of travel by sea may exclude them spreading disease further. Similarly, we analyzed commercial air travel data from 2012, which may not reflect forthcoming patterns of travel in 2014. While we found a highly consistent seasonal pattern of travel between the United States and chikungunya indigenous areas of the Caribbean in earlier years (analyses not shown), travel behaviors this year could be influenced by evolving news of chikungunya in the media. We also relied on accurate vector surveillance data for *Aedes aeygpti *and *albopictus*to identify areas at risk of potential autochthonous transmission. While such vector surveillance has limitations, we used contemporary data reported by the U.S. Centers for Disease Control and Prevention as of January 2014.^13^ Finally, the environmental factors necessary to support autochthonous transmission of chikungunya are complex and influenced not only by the type of vector, but also chikungunya virus characteristics. The climatic conditions required for efficient viral transmission are still under investigation; however, it is likely that warmer temperatures are more favorable. Therefore climatic conditions that evolve over the next several months will likely play a significant role in either hindering or supporting autochthonous transmission of chikungunya.

At a time when locally acquired cases of dengue (also transmitted by *Aedes aeygpti *and *albopictus *mosquitoes) have recently been reported in southern regions of the United States,^31^^-^ 35 our findings highlight the risk for introduction and potential autochthonous transmission of chikungunya virus in selected areas of the country. The effectiveness and efficiency of interventions to mitigate these risks could be optimized through a combination of public education, early detection by medical providers, and the strategic use of public health resources in areas of greatest risk.

## Correspondence

Kamran Khan: khank@smh.ca

## Author Contributions

Kamran Khan and Isaac Bogoch jointly developed the design of the study, oversaw the completion of all analyses, and produced the first draft of the manuscript. Jennifer Miniota, Wei Hu, and Adrian Nicolucci conducted reviews of the literature, performed all transportation and spatial analyses, created figures and cartograms, and edited the final version of the manuscript. John Brownstein contributed epidemiological data pertaining to chikungunya in the Caribbean and made significant content contributions and edits to the final manuscript. Marisa Creatore, Martin Cetron and Annelies Wilder-Smith made significant content contributions to the initial draft of the manuscript and edits to the final draft of the manuscript.

## References

[1] Tomasello D, Schlagenhauf P. Chikungunya and dengue autochthonous cases in Europe, 2007-2012. Travel Med Infect Dis. 2013 Sep-Oct;11(5):274-84. PubMed PMID:23962447. 23962447 10.1016/j.tmaid.2013.07.006

[2] Enserink M. Infectious diseases. Chikungunya: no longer a third world disease. Science. 2007 Dec 21;318(5858):1860-1. PubMed PMID:18096785. 18096785 10.1126/science.318.5858.1860

[3] Reiter P, Fontenille D, Paupy C. Aedes albopictus as an epidemic vector of chikungunya virus: another emerging problem? Lancet Infect Dis. 2006 Aug;6(8):463-4. PubMed PMID:16870524. 16870524 10.1016/S1473-3099(06)70531-X

[4] Powers AM, Logue CH. Changing patterns of chikungunya virus: re-emergence of a zoonotic arbovirus. J Gen Virol. 2007 Sep;88(Pt 9):2363-77. PubMed PMID:17698645. 17698645 10.1099/vir.0.82858-0

[5] Borgherini G, Poubeau P, Staikowsky F, Lory M, Le Moullec N, Becquart JP, Wengling C, Michault A, Paganin F. Outbreak of chikungunya on Reunion Island: early clinical and laboratory features in 157 adult patients. Clin Infect Dis. 2007 Jun 1;44(11):1401-7. PubMed PMID:17479933. 17479933 10.1086/517537

[6] Sissoko D, Moendandze A, Malvy D, Giry C, Ezzedine K, Solet JL, Pierre V. Seroprevalence and risk factors of chikungunya virus infection in Mayotte, Indian Ocean, 2005-2006: a population-based survey. PLoS One. 2008 Aug 26;3(8):e3066. PubMed PMID:18725980. 18725980 10.1371/journal.pone.0003066 PMC2518850

[7] Appassakij H, Khuntikij P, Kemapunmanus M, Wutthanarungsan R, Silpapojakul K. Viremic profiles in asymptomatic and symptomatic chikungunya fever: a blood transfusion threat? Transfusion. 2013 Oct;53(10 Pt 2):2567-74. PubMed PMID:23176378. 23176378 10.1111/j.1537-2995.2012.03960.x

[8] US Centers for Diease Control and Prevention. First reports of Chikungunya in Western Hemisphere. US Centers for Diease Control and Prevention, 2013

[9] Reiskind MH, Pesko K, Westbrook CJ, Mores CN. Susceptibility of Florida mosquitoes to infection with chikungunya virus. Am J Trop Med Hyg. 2008 Mar;78(3):422-5. PubMed PMID:18337338. 18337338 PMC2573391

[10] Gibney KB, Fischer M, Prince HE, Kramer LD, St George K, Kosoy OL, Laven JJ, Staples JE. Chikungunya fever in the United States: a fifteen year review of cases. Clin Infect Dis. 2011 Mar 1;52(5):e121-6. PubMed PMID:21242326. 21242326 10.1093/cid/ciq214

[11] US Centers for Diease Control and Prevention. The Yellow Book: CDC Health Information for International Travel. US Centers for Diease Control and Prevention, 2014

[12] United Nations. Composition of macro geographical (continental) regions, geographical sub-regions, and selected economic and other groupings. United Nations Statistical Division, 2014

[13] US Centers for Diease Control and Prevention. Chikungunya: Information for vector control programs. US Centers for Diease Control and Prevention, 2014

[14] WeatherBase. United States of America - Weather Averages. WeatherBase, 2014

[15] Ruiz-Moreno D, Vargas IS, Olson KE, Harrington LC. Modeling dynamic introduction of Chikungunya virus in the United States. PLoS Negl Trop Dis. 2012;6(11):e1918. PubMed PMID:23209859. 23209859 10.1371/journal.pntd.0001918 PMC3510155

[16] Charrel RN, de Lamballerie X, Raoult D. Seasonality of mosquitoes and chikungunya in Italy. Lancet Infect Dis. 2008 Jan;8(1):5-6. PubMed PMID:18156081. 18156081 10.1016/S1473-3099(07)70296-7

[17] Tilston N, Skelly C, Weinstein P. Pan-European Chikungunya surveillance: designing risk stratified surveillance zones. Int J Health Geogr. 2009 Oct 31;8:61. PubMed PMID:19878588. 19878588 10.1186/1476-072X-8-61 PMC2776014

[18] Fischer D, Thomas SM, Suk JE, Sudre B, Hess A, Tjaden NB, Beierkuhnlein C, Semenza JC. Climate change effects on Chikungunya transmission in Europe: geospatial analysis of vector's climatic suitability and virus' temperature requirements. Int J Health Geogr. 2013 Nov 12;12:51. PubMed PMID:24219507. 24219507 10.1186/1476-072X-12-51 PMC3834102

[19] Greer A, Ng V, Fisman D. Climate change and infectious diseases in North America: the road ahead. CMAJ. 2008 Mar 11;178(6):715-22. PubMed PMID:18332386. 18332386 10.1503/cmaj.081325 PMC2263103

[20] Tatem AJ, Hay SI, Rogers DJ. Global traffic and disease vector dispersal. Proc Natl Acad Sci U S A. 2006 Apr 18;103(16):6242-7. PubMed PMID:16606847. 16606847 10.1073/pnas.0508391103 PMC1435368

[21] Sutherst RW. Global change and human vulnerability to vector-borne diseases. Clin Microbiol Rev. 2004 Jan;17(1):136-73. PubMed PMID:14726459. 14726459 10.1128/CMR.17.1.136-173.2004 PMC321469

[22] Lanciotti RS, Valadere AM. Transcontinental movement of Asian genotype chikungunya virus. Emerging Infectious Diseases 2014;20(8): http://dx.doi.org/10.3201/eid2008.140268 10.3201/eid2008.140268 PMC4111183 25076384

[23] Vega-Rua A, Zouache K, Caro V, Diancourt L, Delaunay P, Grandadam M, Failloux AB. High efficiency of temperate Aedes albopictus to transmit chikungunya and dengue viruses in the Southeast of France. PLoS One. 2013;8(3):e59716. PubMed PMID:23527259. 23527259 10.1371/journal.pone.0059716 PMC3601061

[24] Liu-Helmersson J, Stenlund H, Wilder-Smith A, Rocklov J. Effects of diurnal temperature variations on global dengue epidemic potential. PLoS ONE 2014;In Print.

[25] Schuffenecker I, Iteman I, Michault A, Murri S, Frangeul L, Vaney MC, Lavenir R, Pardigon N, Reynes JM, Pettinelli F, Biscornet L, Diancourt L, Michel S, Duquerroy S, Guigon G, Frenkiel MP, Bréhin AC, Cubito N, Desprès P, Kunst F, Rey FA, Zeller H, Brisse S. Genome microevolution of chikungunya viruses causing the Indian Ocean outbreak. PLoS Med. 2006 Jul;3(7):e263. PubMed PMID:16700631. 16700631 10.1371/journal.pmed.0030263 PMC1463904

[26] Tsetsarkin KA, Vanlandingham DL, McGee CE, Higgs S. A single mutation in chikungunya virus affects vector specificity and epidemic potential. PLoS Pathog. 2007 Dec;3(12):e201. PubMed PMID:18069894. 18069894 10.1371/journal.ppat.0030201 PMC2134949

[27] Bordi L, Carletti F, Castilletti C, Chiappini R, Sambri V, Cavrini F, Ippolito G, Di Caro A, Capobianchi MR. Presence of the A226V mutation in autochthonous and imported Italian chikungunya virus strains. Clin Infect Dis. 2008 Aug 1;47(3):428-9. PubMed PMID:18605910. 18605910 10.1086/589925

[28] Horwood P, Bande G, Dagina R, Guillaumot L, Aaskov J, Pavlin B. The threat of chikungunya in Oceania. Western Pac Surveill Response J. 2013 Apr-Jun;4(2):8-10. PubMed PMID:24015365. 24015365 10.5365/WPSAR.2013.4.2.003 PMC3762969

[29] Wu D, Zhang Y, Zhouhui Q, Kou J, Liang W, Zhang H, Monagin C, Zhang Q, Li W, Zhong H, He J, Li H, Cai S, Ke C, Lin J. Chikungunya virus with E1-A226V mutation causing two outbreaks in 2010, Guangdong, China. Virol J. 2013 Jun 2;10:174. PubMed PMID:23725047. 23725047 10.1186/1743-422X-10-174 PMC3691762

[30] Kumar A, Mamidi P, Das I, Nayak TK, Kumar S, Chhatai J, Chattopadhyay S, Suryawanshi AR, Chattopadhyay S. A novel 2006 Indian outbreak strain of Chikungunya virus exhibits different pattern of infection as compared to prototype strain. PLoS One. 2014;9(1):e85714. PubMed PMID:24465661. 24465661 10.1371/journal.pone.0085714 PMC3896419

[31] Adalja AA, Sell TK, Bouri N, Franco C. Lessons learned during dengue outbreaks in the United States, 2001-2011. Emerg Infect Dis. 2012 Apr;18(4):608-14. PubMed PMID:22469195. 22469195 10.3201/eid1804.110968 PMC3309700

[32] Bouri N, Sell TK, Franco C, Adalja AA, Henderson DA, Hynes NA. Return of epidemic dengue in the United States: implications for the public health practitioner. Public Health Rep. 2012 May-Jun;127(3):259-66. PubMed PMID:22547856. 22547856 10.1177/003335491212700305 PMC3314069

[33] Effler PV, Pang L, Kitsutani P, Vorndam V, Nakata M, Ayers T, Elm J, Tom T, Reiter P, Rigau-Perez JG, Hayes JM, Mills K, Napier M, Clark GG, Gubler DJ. Dengue fever, Hawaii, 2001-2002. Emerg Infect Dis. 2005 May;11(5):742-9. PubMed PMID:15890132. 15890132 10.3201/eid1105.041063 PMC3320380

[34] Radke EG, Gregory CJ, Kintziger KW, Sauber-Schatz EK, Hunsperger EA, Gallagher GR, Barber JM, Biggerstaff BJ, Stanek DR, Tomashek KM, Blackmore CG. Dengue outbreak in Key West, Florida, USA, 2009. Emerg Infect Dis. 2012 Jan;18(1):135-7. PubMed PMID:22257471. 22257471 10.3201/eid1801.110130 PMC3310087

[35] Ramos MM, Mohammed H, Zielinski-Gutierrez E, Hayden MH, Lopez JL, Fournier M, Trujillo AR, Burton R, Brunkard JM, Anaya-Lopez L, Banicki AA, Morales PK, Smith B, Muñoz JL, Waterman SH. Epidemic dengue and dengue hemorrhagic fever at the Texas-Mexico border: results of a household-based seroepidemiologic survey, December 2005. Am J Trop Med Hyg. 2008 Mar;78(3):364-9. PubMed PMID:18337327. 18337327

